# Radiolabeled EGFR TKI as predictive imaging biomarkers in NSCLC patients – an overview

**DOI:** 10.3389/fonc.2022.900450

**Published:** 2022-10-12

**Authors:** Eveline Van De Stadt, Maqsood Yaqub, A. A. Jahangir, Harry Hendrikse, Idris Bahce

**Affiliations:** ^1^ Department of Pulmonology, Amsterdam University Medical Centers (UMC), VU University Medical Center, Amsterdam, Netherlands; ^2^ Department of Radiology and Nuclear Medicine, Amsterdam University Medical Centers (UMC), VU University Medical Center, Amsterdam, Netherlands; ^3^ Cancer Center Amsterdam, Amsterdam University Medical Centers (UMC), Amsterdam, Netherlands

**Keywords:** NSCLC, EGFR TKI, PET/CT, radiolabeled EGFR TKI, molecular imaging

## Abstract

Non-small cell lung cancer (NSCLC) has one of the highest cancer-related mortality rates worldwide. In a subgroup of NSCLC, tumor growth is driven by epidermal growth factor receptors (EGFR) that harbor an activating mutation. These patients are best treated with EGFR tyrosine kinase inhibitors (EGFR TKI). Identifying the EGFR mutational status on a tumor biopsy or a liquid biopsy using tumor DNA sequencing techniques is the current approach to predict tumor response on EGFR TKI therapy. However, due to difficulty in reaching tumor sites, and varying inter- and intralesional tumor heterogeneity, biopsies are not always possible or representative of all tumor lesions, highlighting the need for alternative biomarkers that predict tumor response. Positron emission tomography (PET) studies using EGFR TKI-based tracers have shown that EGFR mutational status could be identified, and that tracer uptake could potentially be used as a biomarker for tumor response. However, despite their likely predictive and monitoring value, the EGFR TKI-PET biomarkers are not yet qualified to be used in the routine clinical practice. In this review, we will discuss the currently investigated EGFR-directed PET biomarkers, elaborate on the typical biomarker development process, and describe how the advances, challenges, and opportunities of EGFR PET biomarkers relate to this process on their way to qualification for routine clinical practice.

## 1 Introduction

Lung cancer is one of the most prevalent cancer types worldwide (1). Lung cancer accounts for approximately 22% of all cancer-related mortality, emphasizing that lung cancer is not only a highly prevalent cancer type, but also one of the deadliest (1). For decades, the standard of care treatment for advanced stage non-small cell lung cancer (NSCLC) was only chemotherapy (2–5). The introduction of tyrosine kinase inhibitors (TKI) directed against the epidermal growth factor receptor (EGFR), an oncogenic driver pathway promoting cell growth and division, led to a shift in the treatment paradigm of EGFR mutation positive NSCLC, and, ultimately to an acceleration of the development of targeted therapies against other oncogenic driver mutation targets (2–5). Wild type EGFR activation is ligand-dependent, i.e., the EGFR kinase function only activates if an EGF ligand is bound at the extracellular binding site of the receptor (6). However, with activating mutations in the EGFR kinase domain, activation occurs in the absence of a ligand, leading to tumor cell proliferation and growth (6). EGFR TKIs bind with high affinity at the kinase domain of the mutated EGFR and block its function (6, 7). As a result, patients harboring activating EGFR mutations achieve higher tumor responses on EGFR TKI than on conventional chemotherapy (2–4, 8).

The iPASS trial was the first trial that clearly showed the superior clinical efficacy of EGFR TKI as compared to conventional chemotherapy. In this study, Mok et al. demonstrated that the first-generation EGFR TKI gefitinib achieved a higher progression-free survival (PFS) in the intention-to-treat population (HR 0.74; 95%CI 0.65 to 0.85; P<0.001) (3). Many other first-line phase 3 clinical studies using the first-generation EGFR TKI gefitinib or erlotinib, showed comparable results (2, 4, 9, 10). In contrast to the first-generation TKIs, the second-generation TKIs afatinib and dacomitinib were characterized by an irreversible binding of the TKI to the EGFR kinase domain and by multi-kinase targeting (5, 10–15). These second-generation TKIs had possibly a superior efficacy as compared to first-generation TKI at the cost of slightly higher toxicities (10, 16). The third-generation TKI osimertinib was primarily designed to target the secondary resistance mutation T790M (17–21). In the AURA3 trial, patients with T790M secondary mutations, occurring as resistance mutations on an initial treatment with gefitinib or erlotinib, were randomized between osimertinib versus conventional chemotherapy (17). Osimertinib showed superior PFS (10.1m vs. 4.4m; HR 0.30; 95%CI 0.23 to 0.41; P<0.001). The objective response rate was also significantly better with osimertinib (71%; 95% CI, 65 to 76) than with chemotherapy (31%; 95% CI, 24 to 40) (OR 5.39; 95%CI 3.47 to 8.48; P<0.001) (17). Surprisingly, osimertinib also performed above expectations as a first-line therapy. In the FLAURA study, treatment-naïve EGFR mutation positive patients were randomized to osimertinib versus a first-generation EGFR TKI (22). Osimertinib showed superior PFS (18.9m vs. 10.2m; HR 0.46; 95%CI 0.37 to 0.57; P<0.001). In a recent update of the study results, osimertinib also showed OS superiority as compared to the first-generation TKI (38.6m vs. 31.8m; HR 0.80; 95%CI 0.64 to 1.00; P=0.046) (23). These developments illustrate that over the course of approximately a decade, significant advances have been made in the treatment of EGFR mutation positive NSCLC, and that the identification of these patients is of paramount importance.

Diagnosis through next-generation sequencing of tumor DNA, obtained through a histological biopsy, is the gold standard for identifying tumor EGFR mutations (24). Unfortunately, taking biopsies is invasive, at risk for complications and not always possible due to difficult to reach tumor sites. Also, biopsies may not always be representative for all the tumor lesions due to varying intra- and interlesional heterogeneity, this may especially be of importance when resistance occurs and mapping the residual sensitivity for TKI treatment is needed (24). To overcome these limitations new biomarkers have been investigated. Liquid biopsies are ever more used in situations when representative tumor biopsies cannot be obtained. Even though the current sensitivity of liquid biopsies is approximately 70% with specificities above 90%, not all patients can be diagnosed using liquid biopsies alone (25, 26). Also, liquid biopsies do not address the limitation of tumor heterogeneity. Alternatively, in recent years, imaging studies using radiolabeled EGFR TKI have shown that PET could potentially be of value for identifying EGFR mutation positive patients and predicting tumor sensitivity to EGFR TKI (27–31).

In this review, we will discuss the current EGFR-directed PET tracers that have been investigated in EGFR mutated NSCLC. The special focus will lie with radiolabeled EGFR TKI: inertly labeled EGFR TKI used as a PET tracer in NSCLC patients. In addition, we will discuss the framework of the PET biomarker development process, highlighting the different contexts of use to better elucidate the stage in which these EGFR TKI PET biomarkers are at. We will describe the challenges, but also the recent advances and opportunities that could help EGFR PET on its path to generating qualified predictive biomarkers for clinical use.

## 2 Current EGFR PET biomarkers

### 2.1 PET biomarker background

PET is a molecular imaging technique, widely in use in the staging and treatment monitoring schedules in cancer patients. A radioactively labeled compound used as a tracer, which is expected to accumulate at the site of a specific target in the tumor lesion, is injected into the body and its distribution is then imaged. When using a validated tracer, its accumulation in the tumor and other sites is expected to be sensitive and quantifiable. The tracer accumulation or the so-called tracer uptake can be measured using different metrics, which can serve as biomarkers.

In general, a biomarker is a measurable indicator of a biological process and in case of PET imaging, this can be a measure derived from the tracer uptake in tumors or in healthy tissues, e.g., the Standardized Uptake Value (SUV) or the Distribution Volume (V_T_).

**Figure 1:**
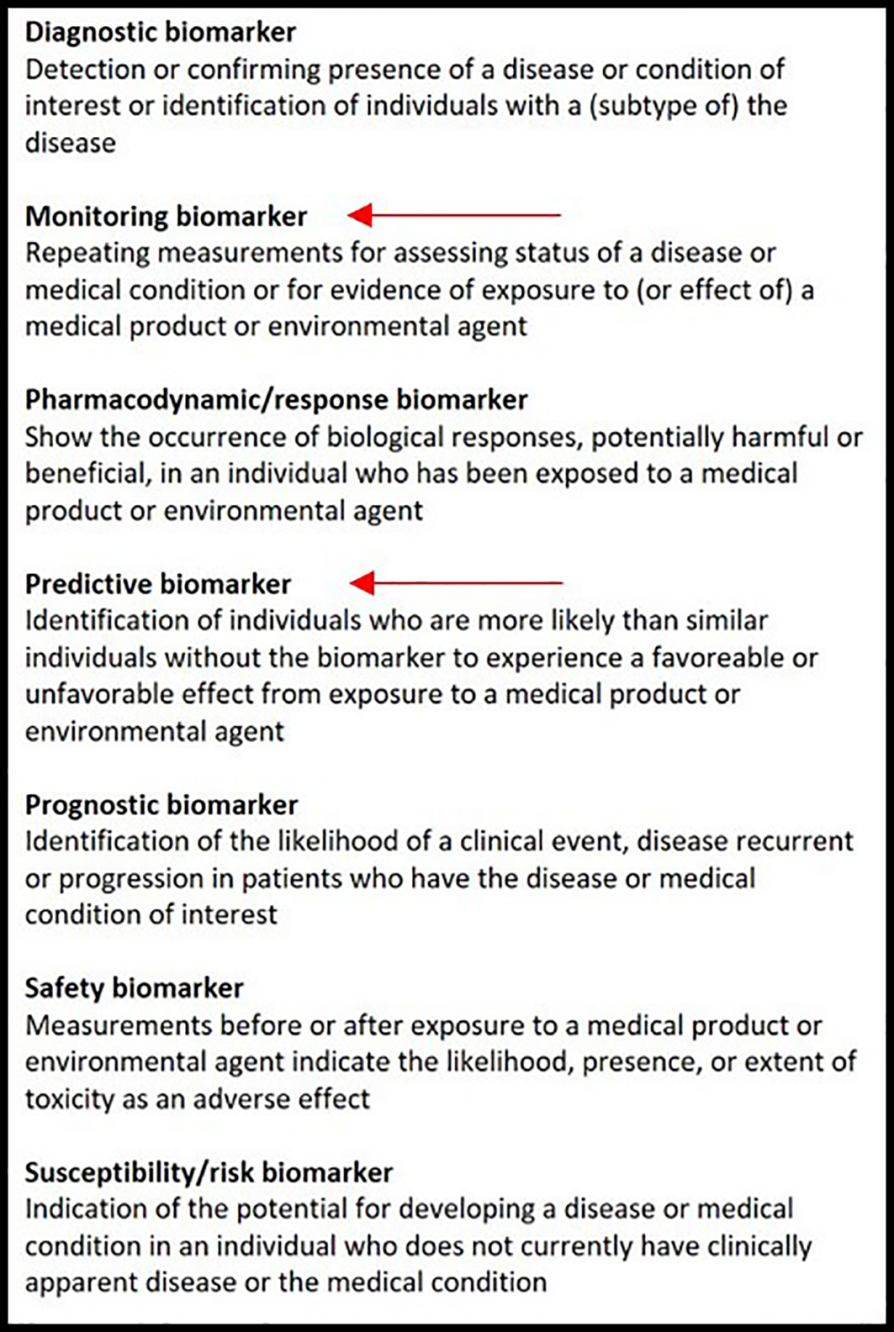
The biomarker classification according to the BEST biomarker categories. The red arrows indicate in which category EGFR-directed PET tracers could be included.

Also, depending on their aims, biomarkers will have different ‘contexts of use’. The evidence that is necessary to support qualification towards clinical practice is dependent on the specific context of use. The FDA Qualification Framework recommends categorizing biomarkers using the BEST biomarker categories according to their aims, as described in **Figure 1** (32).

Considering EGFR, PET should provide a *predictive biomarker*, which is most relevant for the clinical practice. The presence of common EGFR mutations (i.e., exon19 deletions, exon21 L858R) are highly predictive for response to TKI therapy; however, in case of uncommon mutations, less is known regarding their clinical relevance and tumor TKI responses may vary greatly between different uncommon mutations. A predictive PET biomarker would therefore be most interesting.

EGFR directed PET biomarkers will *de facto* never be able to diagnose an activating EGFR mutation, as this requires tumor DNA sequencing on tumor tissue or liquid biopsies. Therefore, a PET imaging biomarker could never be a *diagnostic biomarker* that replaces DNA sequencing. On the other hand, PET imaging biomarkers could very well become qualified as predictive biomarkers to predict tumor sensitivity to EGFR TKI as mentioned before.

A *monitoring biomarker* is also of interest, as all tumors eventually develop resistance to EGFR TKI, in which case it could be of clinical importance to know whether lesions or parts of lesions remain TKI sensitive to decide whether TKI should be continued beyond progression.

The current PET biomarkers can be categorized into 2 categories, i.e., those based on non-EGFR-directed tracers and those that are derived from EGFR TKI-based tracers.

### 2.2 Non-EGFR PET biomarkers in EGFR mutated NSCLC

The most widely-used tracer is ^18^F-fluorodeoxyglucose (^18^F-FDG), a radioactive analogue to glucose, that can quantify metabolic activity. In the past decade, many clinical studies attempted to establish the role of ^18^F-FDG in evaluating the EGFR mutational status (33). A meta-analysis by Du et al. looked at studies that compared the lesional maximum of standardized uptake value (SUV_max_) of ^18^F-FDG uptake between wild-type and mutant EGFR and evaluated its value for predicting the EGFR status in NSCLC patients (33). In 15 studies (3574 patients), the pooled sensitivity and specificity was found to be low. The authors concluded that ^18^F-FDG based SUV_max_ should be used with caution when predicting EGFR mutations in NSCLC (33). However, new studies are exploring the potential outcome of radiomics and artificial intelligence (AI) algorithms as biomarkers to assess the predictive capacity of ^18^F-FDG PET. For example, Yin et al. demonstrated in a training data set of 198 NSCLC patients with a testing data set of 103 patients that their algorithm could predict EGFR mutations automatically with a ROC-AUC of 0.84 (95% CI, 0.75–0.90) (34). These developments may indicate an increasing role for radiomics and AI as new ^18^F-FDG based biomarkers in the future, albeit, these algorithms need optimization and validation using larger cohorts.

In recent years, 3-deoxy-3-^18^F-fluorothymidine (^18^F-FLT) PET scans have generated interest in oncology. As opposed to ^18^F-FDG, ^18^F-FLT PET reflects cell proliferation (10, 35). This tracer is trapped intracellularly in the S-phase of the cell cycle (35). Elevated ^18^F-FLT uptake of lesions could therefore be indicative of tumor cell proliferation and treatment-resistance. This supports the notion that ^18^F-FLT could serve to generate treatment monitoring biomarkers. Indeed, studies using ^18^F-FLT in EGFR mutation positive NSCLC have shown that a decrease of ^18^F-FLT uptake in tumor lesions is associated with response to EGFR TKI treatment (10, 36, 37). As ^18^F-FLT is nonspecific to EGFR mutations, the validation of ^18^F-FLT-based monitoring biomarkers could be of interest for many cancer types as well.

Other non-EGFR PET tracers that have been investigated in EGFR mutation positive NSCLC patients, are ^11^C-choline and *O*-(2-[^18^F]fluoroethyl)-L-tyrosine(^18^F-FET). ^11^C-choline, a tracer mainly used in diagnostics of prostate cancer, is a component of phospholipids in the cell membrane (38). Phosphorylation of choline is upregulated in cancers through choline-kinase (38). Although ^11^C-choline PET is used in the routine practice in other cancer types, results in NSCLC are discouraging (39–41). ^18^F-FET has been used in diagnostics of brain tumors, including brain metastases of NSCLC, however, no studies were published on ^18^F-FET in extracranial NSCLC tumors (42, 43).

### 2.3 EGFR PET biomarkers

#### 2.3.1 Characteristics of EGFR PET tracers

For radiolabeling target-specific drugs such as EGFR TKI, the characteristics of the radionuclide that is used for labeling needs to be aligned with the pharmacokinetic properties of the parent compound. For example, using radionuclides with long-lived isotopes such as zirconium-89 (t_1/2_ 78 hours) are best suited to label large molecules with slow pharmacokinetics like monoclonal antibodies, e.g., ^89^Zr-cetuximab, however, inappropriate for labeling EGFR TKI. Since EGFR TKI are small molecules with relatively fast pharmacokinetics, i.e., fast target binding and rapid clearance from the circulation, using short-lived isotopes such as carbon-11 (t½ 20 min) or fluorine-18 (t½ 110 min) is more appropriate.

Also, instead of adding the radionuclide on the parent compound, substituting an existing carbon or fluorine atom of the TKI molecule will maintain the original pharmacokinetic (PK) behavior of the TKI resulting in a tracer that is equally specific as the original TKI. The choice whether carbon-11 or fluorine-18 is used for this inert substitution is based on the chemical structure of the parent compound (27, 31, 44).

**Figure 2:**
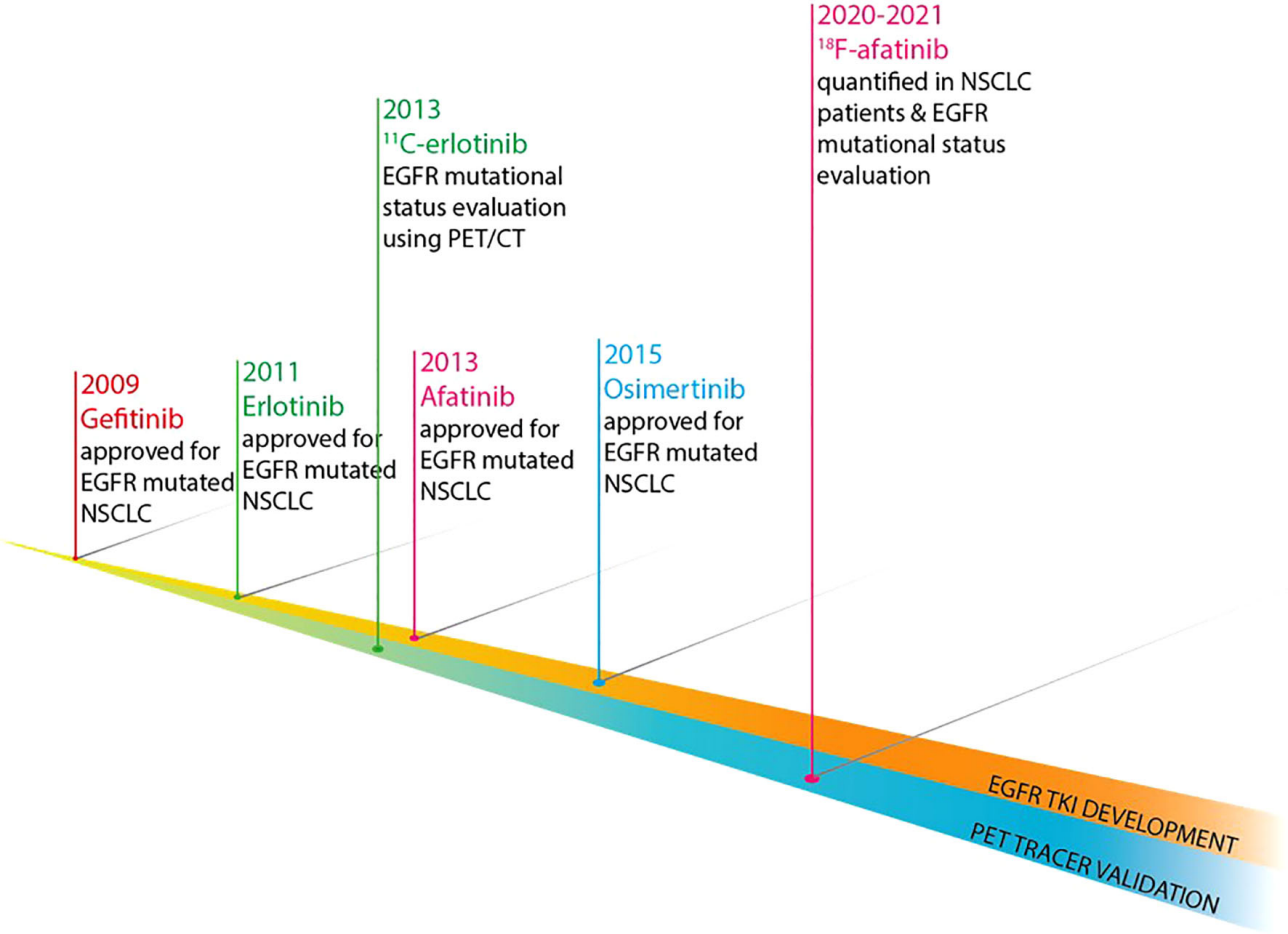
Development timeline of EGFR TKI and their respective EGFR-directed PET tracers. .

Although tracers based on EGFR TKI that are in clinical use, when labeled inertly, provide the best PK behavior metrics to investigate tumor sensitivity to the respective TKI, the development of such tracers is inherently delayed, as clinical safety and efficacy data of the parent TKI need to be established. Moreover, the fast development of subsequent generations of TKI could disrupt the development of early generation TKI tracers and make them redundant. To illustrate this, a timeline indicating the approval of the 3 generations of EGFR TKI used in the clinical and their tracer counterparts is shown in **Figure 2**.

Clinical PET studies are not only being performed using EGFR PET tracers based on EGFR TKI, but also on tracers without treatment analogue. Many of these tracers without direct treatment analogue have been specifically developed for the purpose of imaging. These tracers, e.g., ^18^F-MPG, ^11^C-PD153035 and ^18^F-IRS, show significant differences amongst themselves in kinetic characteristics, mainly in the binding affinity to the kinase domain (45–47).

#### 2.3.2 Present EGFR TKI PET biomarkers

An overview of published clinical studies using EGFR PET tracers is given in **Table 1**. For ^11^C-erlotinib and ^18^F-afatinib, studies have shown that EGFR mutation positive patients can be identified and that tumor response to treatment using the corresponding EGFR TKI (27, 31) could be predicted using PET biomarkers. This was seen in patients with common and uncommon EGFR mutations. For ^11^C-osimertinib, the clinical studies investigating its predictive value are still ongoing.

**Table 1:** Overview of clinical EGFR TKI PET studies.

Year	Tracer	N	Uptake parameter	Kinetic modeling?	Used as biomarker for EGFR status?	EGFR mutation in studies	Study
2008	^11^C-PD153035	11	SUV	No	No	Exon 19 & exon 21 mutations	Yu et al. (48)
2009	^11^C-PD153035	14	SUV_max_	No	Yes	Exon 19 & exon 21 mutations	Yu et al. (49)
2010	^11^C-PD153035	19	SUV_max_	No	No	Unknown	Liu et al. (50)
2011	^11^C-PD153035	21	SUV_max_	No	No	Unknown	Meng et al. (45)
2011	^11^C-Erlotinib	13	Radioactivity per mL tissue	No	No	Unknown	Memon et al. (51)
2013	^11^C-Erlotinib	10	V_T_	Yes	Yes	Exon 19 del	Bahce et al. (27)
2017	^18^F-IRS	3	SUV_max_	No	Yes	Exon 19 del	Song et al. (47)
2018	^18^F-MPG	75	SUV_max_	No	Yes	Unknown	Sun et al. (46)
2018	^18^F-ODS2004436	20	SUV_ratio_	No	Yes	Unknown	Cochet et al. (52)
2021	^18^F-Afatinib	12	TBR_WB_60-90_	Yes	Yes	Exon 19 deletion, exon 19 L747P insertion, exon 18 G719A point mutation, exon 18 G709T deletion	van de Stadt et al. (30)
2021	^11^C-erlotinib	10	V_T_ & SUV_mean_	Yes	Yes	Exon 19 deletion, L858R point mutation, G719S + S768I mutation, L861Q mutation	Petrulli et al. (53)

The EGFR mutational status as described in the study is shown. SUV, standardized uptake value; V_T_, volume of distribution; TBR_WB_60-90_, tumor-to-whole-blood ratio in the time interval 60-90 minutes post-injection.

**Table 2:** Key tracer targets for each tracer are shown.

Tracer	Key tracer targets	
^11^C-PD153035	EGFR (wild-type and mutations), HER2	(54)
^11^C-erlotinib	1^st^-generation TKI: common EGFR mutations (exon 19del, exon 21 L858R), partly wild-type EGFR, not T790M	(7)
^18^F-IRS	Comparable to 1^st^-generation TKI: common EGFR mutations, no T790M	(48)
^18^F-MPG	Common EGFR mutations, not wild type EGFR, not T790M	(47)
^18^F-ODS2004436	Limited data is publicly available, targets wild type and exon 21 L858R, not T790M	(55, 56)
^18^F-afatinib	2^nd^-generation TKI: common EGFR mutations (exon 19del, exon 21 L858R) + other ERBB family kinases, partly T790M	(57–59)
^11^C-osimertinib	3^rd^-generation TKI: specifically developed for EGFR T790M mutation, common EGFR mutations, also uncommon non-exon20 insertions, not wild type EGFR	(20, 23)

For EGFR PET tracers without treatment analogue, e.g., ^18^F-MPG, ^11^C-PD153035 and ^18^F-IRS, studies have shown that tumor tracer uptake could be quantified and that this was predictive for the presence of an EGFR mutation and for TKI therapy response (45–47). Both ^18^F-IRS and ^11^C-PD153035 showed a close relation between tracer uptake (SUV_max_) and EGFR expression, and for all three tracers a correlation between uptake (SUV_max_) and treatment response was observed (45–47).

The overview in **Table 1**, comprising approximately 200 NSCLC patients, summarizes several study characteristics. When new tracers are introduced, the pharmacokinetic behavior of this tracer needs to be established by performing kinetic modeling. Kinetic modeling allows to better understand the obtained PET images and to quantify the tracer uptake using optimal dynamic parameters of uptake such as ‘Distribution Volume’ (V_T_). For some tracers, this has been performed, as indicated in **Table 1**. In the absence of dynamic uptake parameters, usually simplified static uptake parameters such as ‘Standardized Uptake Values’ (SUVs) are used. For some tracers such as ^11^C-erlotinib and ^18^F-afatinib, the pharmacokinetic modeling has been published and, in these tracers, uptake parameters other than SUV have been suggested (29, 30, 53). In **Table 2**, tracer targets are listed for each tracer.

While this overview highlights the efforts done to investigate and discover the potential of the existing EGFR PET tracers and their biomarkers, it also highlights that data is scarce. From a clinical point of view, the question rises on what would be needed for EGFR PET biomarkers to be able to qualify in the routine clinical practice. To better understand the framework in which such a qualification occurs, we will below elaborate on the typical biomarker development process and how the current state of these tracers and their respective biomarkers relate to this process.

## 3 Challenges and opportunities in the development of EGFR PET biomarkers

### 3.1 Development process of PET biomarkers

**Figure 3:**
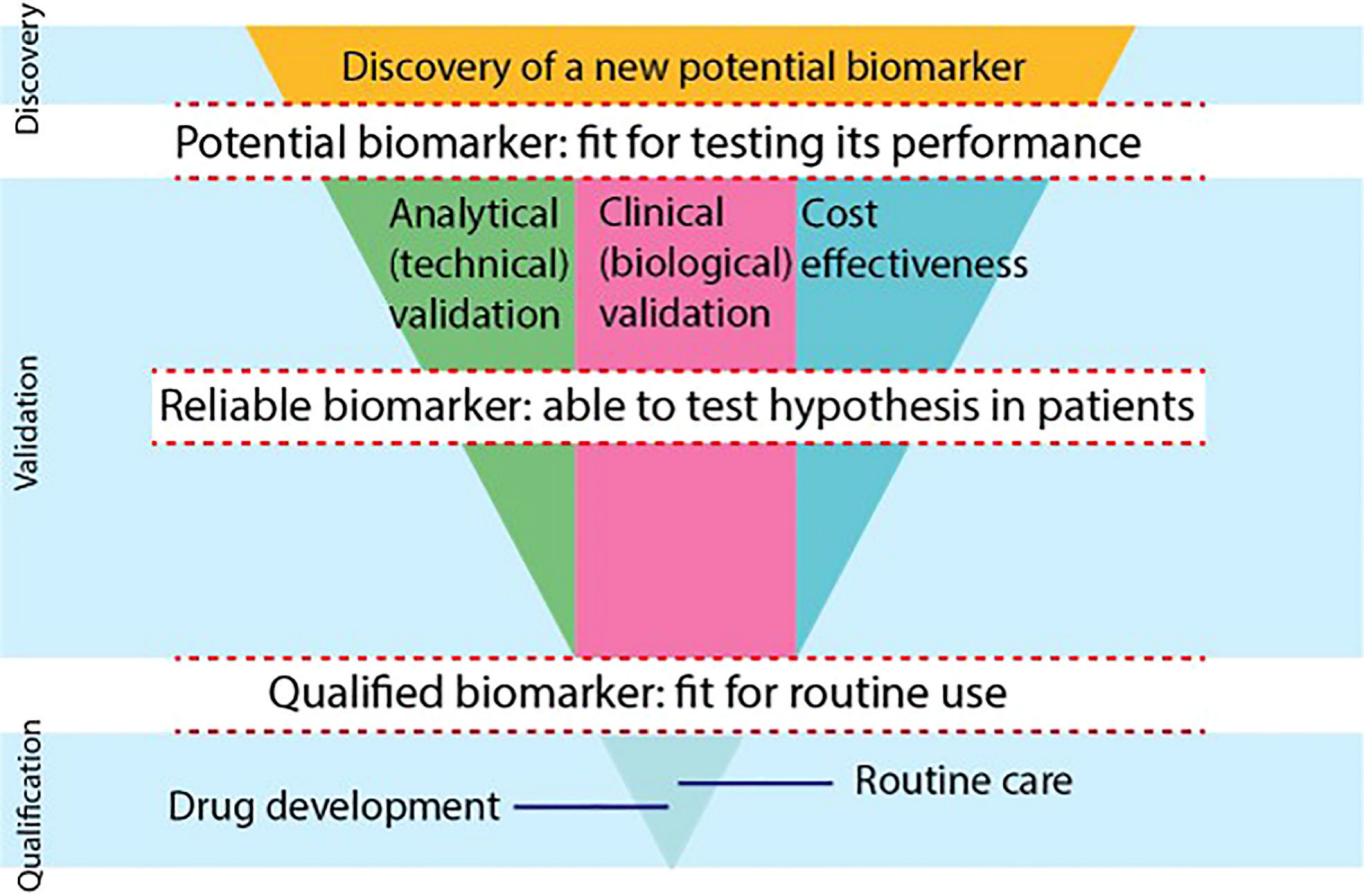
The biomarker development process is shown using a modified scheme, based on the consensus statement on biomarker development of the CRUK and EORTC (59). There are 3 phases of development (discovery, validation, qualification) that biomarkers go through. Biomarkers need to overcome gaps to become potential biomarkers, reliable biomarkers and qualified biomarkers. In the validation phase, 3 separate tracks will be evaluated in parallel and iteratively, i.e., the analytical, clinical and cost-effectiveness track. To be able to use a biomarker in drug development or in routine clinical care, biomarkers need to provide qualification evidence. (*) The FDA Evidentiary Framework provides recommendations that guide the evidence needed to support qualification, bridging the final gap to routine care and drug development in the qualification phase.

To be able to qualify for use in the clinical practice, there are 3 main phases of development that a PET imaging biomarker must transition. See **Figure 3**, which is based on the consensus paper of the CRUK and the EORTC (59). In transitioning from one phase into another, biomarkers need to bridge several gaps. The first gap for a biomarker is to be able to enter the validation phase as a potential biomarker, fit to be tested for performance. In the validation phase, a biomarker needs to proof it is reliable and ‘fit for purpose’. For the development of PET biomarkers, the 3 main validation tracks (analytical, clinical and cost-effectiveness validation) are typically developed in parallel and in an iterative manner. In the qualification phase, sufficient evidence will be needed to support the qualification of a biomarker for a specific context of use in drug development or routine clinical care. support qualification of a biomarker.

### 3.2 EGFR PET biomarker validation challenges

#### 3.2.1 Analytical validation

The analytical validation track evaluates the measures related to biomarker precision, e.g., repeatability, reproducibility and technical bias, and the measures related to biomarker availability in the targeted patient group. The analytical validation, generally, does not consider the clinical utility of the biomarker, however, poor analytical features will hamper the clinical validation and qualification (59).

**Figure 4:**
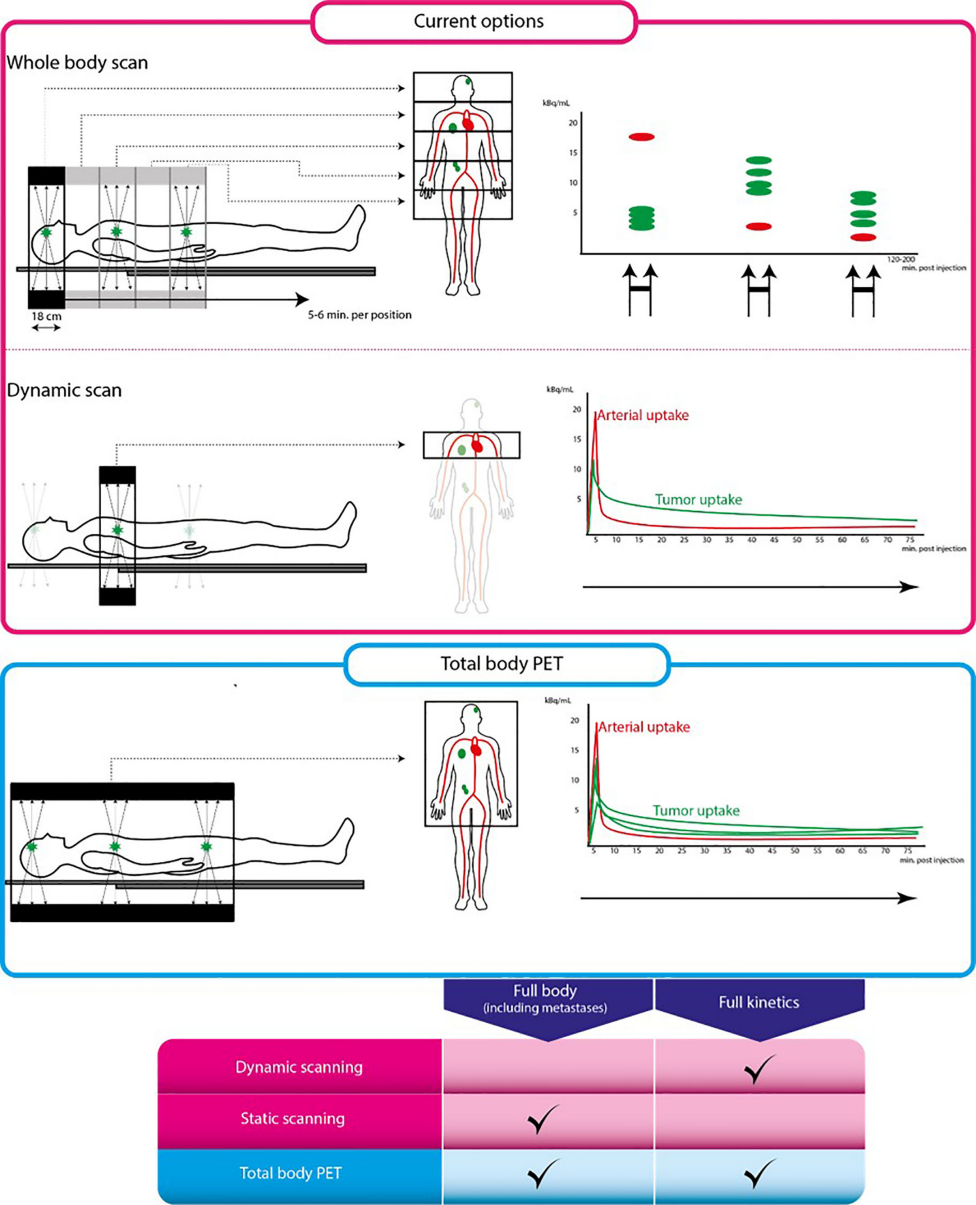
Conventional PET scan versus total body PET scan. From left to right: schematic representation of scan procedure, illustrations on the left are parts of the body that can be scanned using each scanning technique. Illustrations on the right are tracer uptake quantification differences for each technique. The pink box represent conventional PET scanning, the blue box represents total body PET. Table below shows characteristics of each scanning technique. Full kinetics indicates whether quantification using pharmacokinetic modeling is possible using this technique.

Ideally, new EGFR PET tracers for biomarking EGFR that are used in humans will undergo full kinetic modeling. This is an elaborate dynamic PET scanning procedure with arterial blood sampling and measurement of blood radioactivity and blood tracer metabolites. A dynamic PET scan is a continuous scan of 1 section of the body, where both the tumor and a large blood pool or vessel is included in the field of view (FoV), as depicted in **Figure 4**. Since conventional PET scanners have a limited (e.g., 18 cm) FoV, only a small part of the body where the tumor is located will be scanned continuously. The pharmacokinetic behavior over time of the tumor tracer concentration will be measured to produce a time-activity-concentration curve (TAC). Additionally, the radioactivity concentrations of the arterial blood pool over time will be measured to calculate the so-called blood ‘input functions’ using both blood samples from an arterial cannula, and PET image-derived blood pool data. Also, metabolites will be measured repeatedly *via* arterial blood samples to calculate the true parent tracer concentrations over time. Using the TACs, the blood input function and the metabolites data, the pharmacokinetic model that best describes the pharmacokinetic behavior of the tracer in the tumor will be established. This pharmacokinetic model yields various physiologic parameters, which can be used to select the optimal tracer uptake parameter to quantify the tracer uptake. These dynamic uptake parameters are considered the most precise biomarkers for tracer uptake. Only a few EGFR PET tracers such as ^11^C-erlotinib and ^18^F-afatinib have undergone full kinetic modeling.

To evaluate intra-patient repeatability is another step in the analytical validation of a biomarker to assure that biomarkers produce similar results when repeatedly measured in the same circumstances. This has been shown for tumor ^11^C-erlotinib V_T_, however, this crucial step is lacking in many other tracers.

Availability of short-lived EGFR PET tracers is limited due to the short half-life of their radionuclides. For examples, the half-life of carbon-11 is approximately 20 minutes, meaning that the scan must be performed in the same center where the tracer is produced and cannot be exported to other centers. The half-life of fluorine-18 is approximately 5 times longer (t ½ ~110 min), which allows shipping to external not-too-distant centers. Another factor limiting the availability is the scarcity of expertise to apply the complex algorithms used to interpret uptake. In the same vein, dedicated software with intuitive user-friendly interfaces are lacking.

#### 3.2.2 Clinical validation

The clinical validation is a process in which the relationship of a biomarker to a clinical feature is evaluated. Biomarkers are typically linked to biological mechanisms of action at the tumor microenvironment. Ultimately, depending on the context of use, the clinical validation should lead to the identification of biomarkers that benefit clinical outcomes or improve the prevention, screening, staging, diagnosis, therapies, or care of patients (59).

Insights obtained in clinical validation studies will feedback into the analytical validation process in order to further optimize the technical aspect of the biomarker. This positive feedback loop highlights the interdependency between these two tracks. Another time-consuming factor in this (clinical) track is the fact that large, prospective clinical PET studies will only be initiated after analytical validation studies have established the precision and accuracy of the tracer as an EGFR biomarker.

The prompt introduction of new EGFR TKI therapy options and the rapid changes in the standard of care for these patients pose a risk on the EGFR PET tracer development, as most TKI-based tracers have a few years of delay vis-à-vis their therapeutic parents, which can lead to tracers become obsolete. This is highlighted by the timeline depicted in **Figure 2**: approval of afatinib dates back to 2013, whereas research regarding ^18^F-afatinib was first published in 2020, a 7-year delay. In contrast, osimertinib was approved for clinical use in 2015, only 2 years after afatinib entered the market and 5 years before the first publication of ^18^F-afatinib.

#### 3.2.3 Cost-effectiveness

In the cost-effectiveness track, the costs associated with the use of biomarkers need to be assessed. To become a qualified biomarker for clinical use, these costs need to compare favorably to the existing alternative biomarkers such as bio specimen-derived biomarkers, e.g., liquid biopsies. Costs may become lower at a later stage after broad-scaled implementation (59).

The added advantage of the EGFR PET is to evaluate tumor EGFR TKI sensitivity when regular biopsies are not informative enough or for obtaining spatial insights in the tumor TKI sensitivity to guide decision-making. This technique is therefore used in addition to regular biopsy-techniques. Consequently, evaluating the cost-effectiveness for these situations is difficult. With further analytical and clinical optimization supported by upcoming PET technology and improved data processing algorithms, EGFR PET biomarkers hold promise to provide value for their costs. However, at the current stage, no EGFR PET tracer could be considered cost-effective, especially when compared to biopsy-techniques already widely-used in clinical practice.

### 3.3 Opportunities

The clinical implementation of EGFR PET biomarkers have been limited by the abovementioned challenges, however, recent developments in emerging new technologies are promising to help the biomarker validation process. Although technological advancements may seem to mainly benefit the technical validation and cost-effectiveness tracks, these optimizations feedback positively to the clinical track as well and therefore improve the full validation process. One of the developments that will advance the validation of EGFR PET biomarkers will be the large-scaled introduction of the so-called ‘total body PET’.

#### 3.3.1 Total body PET

The total body PET scanner refers to a new generation of commercially available PET-CT scanners that have a much larger axial FoV as compared to conventional state-of-the-art PET-CT systems with an axial FOV of less than 20cm. These new large-FoV PET-CT systems achieve ultra-high (40-to-200-fold higher) sensitivity and allow to visualize and quantify tracer uptake in all major internal organs in the body simultaneously (60–64). This provides numerous new imaging opportunities for patient care and research, since these total body PET-CT scanners will speed up the validation of EGFR PET biomarkers by optimizing their analytical validation and by supporting the clinical validation.

One of the advantages of the ultra-high sensitivity will be the possibility to use lower amounts of radioactivity per tracer injection, which will enable to lower the radiation burden to the patients (60, 64). This could make EGFR PET imaging biomarkers more suited for therapy monitoring through performing multiple PET-CT scans longitudinally.

For static tracer uptake parameters such as SUV, another advantage of the ultra-high sensitivity will be the shorter scan durations (currently 30-40 min per ^18^F-FDG PET scan), which in turn will improve patient comfort. The optimal scan duration per EGFR tracer on the total body PET-CT scanner is not clear yet, but this could be as short as 20 seconds (a breath-hold) for some tracers. Short acquisition times could also significantly decrease possible *partial volume effects* caused by smearing the PET signal by the movement of small lesions, e.g., due to breathing-motions (60, 64). Also, this will reduce co-registration mismatch of the PET and CT data, e.g., because of patients moving on the scanner while scanning, which generates artefacts in the reconstructed PET data due to faulty CT-attenuation correction (60, 64). These improvements will increase the resolution and precision of the PET biomarkers, broadening their applicability.

For dynamic tracer uptake parameters, combining the large-axial FOV and the ultra-high specificity of the PET-CT system could greatly improve biomarker specificity, repeatability, and reproducibility. As compared to static PET studies, using dynamic PET studies allows to better characterize the pharmacokinetic (PK) behavior of short-lived tracers by generating dynamic tracer uptake parameters (i.e., biomarkers) that are more target specific and accurate than simplified static parameters (60–64). Typically, the limited axial FoV of the conventional PET-CT systems limits most dynamic scans to single organ studies. Also, for dynamic kinetic analysis a so-called ‘arterial input function’ is needed to describe the bioavailability of the radiotracer in blood. The total body PET-CT, covering all major organs and arterial blood pools (eliminating the need for an arterial cannula) could not only dynamically scan most tumor lesions and all major organs at once but could also provide a reliable image-based arterial input function, non-invasively and automatically, which could generate easily-accessible dynamic uptake parameters with higher specificity and precision (60, 64). Also, the large-FOV coverage will generate new insights on biodistribution in healthy organs, which may open avenues for discovering new PET biomarkers to predict toxicity or biomarkers to guide drug dosing.

Using the total body PET-CT would allow to address many of the analytical validation steps in a single PET study, while this would require many studies using the conventional PET system. Speeding up the analytical validation would significantly fasten the clinical validation as well. As less patients would be needed in the various validation steps of a biomarker, this would ultimately be more cost-effective, through shortening the delay between the introduction of a new EGFR TKI and its validation testing. As most of the tumor lesions, all the major organs and a significant part of the blood pool will be included in the dynamic scans, more comprehensive and automatable scanning and data processing algorithms will be developed. With such algorithms, uptake parameters will be produced more easily, and may require less effort from the PET physics personnel.

#### 3.3.2 Further optimizations

With the advent of new PET technologies and improved data processing algorithms, radiolabeling new EGFR TKI could be of interest for pharmaceutical companies to learn about the biodistribution and PK behavior of their new EGFR TKI therapies at an early stage of development. For example, variations in the brain tissue penetration and uptake of TKI in the brain metastases could be of interest as there is quite some variability in the brain penetration of different TKIs (65). Also, blocking studies could be used to explore the optimal dosing to saturate all targets to support the optimal dosing strategy of a TKI (66, 67). The analytical validation associated with these pharmacological drug development projects could support the clinical validation effort as well.

## 4 Conclusion

The use of EGFR TKI PET tracers can generate predictive biomarkers to identify and monitor patients who will respond to EGFR TKI therapies. Current EGFR TKI tracer biomarkers are still in a validation phase, where clinical and analytical improvements loop back iteratively. New developments such as the availability of large-FoV total body PET systems and more improved data processing algorithms can optimize the EGFR TKI PET biomarker validation process. Nevertheless, more evidence is needed for their qualification as predictive and monitoring biomarkers in drug development and routine clinical practice.

## Author contributions

MY: supervision, edit and rewriting. HH: supervision, edit and rewriting. IB: supervision, edit and rewriting. All authors contributed to the article and approved the submitted version.

## Conflict of interest

The authors declare that the research was conducted in the absence of any commercial or financial relationships that could be construed as a potential conflict of interest.

## Publisher’s note

All claims expressed in this article are solely those of the authors and do not necessarily represent those of their affiliated organizations, or those of the publisher, the editors and the reviewers. Any product that may be evaluated in this article, or claim that may be made by its manufacturer, is not guaranteed or endorsed by the publisher.

## References

[1] Siegel RL Miller KD Fuchs HE Jemal A . Cancer statistics, 2021. CA Cancer J Clin (2021) 71(1):7–33. doi: 10.3322/caac.21654 33433946

[2] Maemondo M Inoue A Kobayashi K Sugawara S Oizumi S Isobe H . Gefitinib or chemotherapy for non-small-cell lung cancer with mutated EGFR. N Engl J Med (2010) 362(25):2380–8. doi: 10.1056/NEJMoa0909530 20573926

[3] Mok TS Wu YL Thongprasert S Yang CH Chu DT Saijo N . Gefitinib or carboplatin-paclitaxel in pulmonary adenocarcinoma. N Engl J Med (2009) 361(10):947–57. doi: 10.1056/NEJMoa0810699 19692680

[4] Rosell R Carcereny E Gervais R Vergnenegre A Massuti B Felip E . Erlotinib versus standard chemotherapy as first-line treatment for European patients with advanced EGFR mutation-positive non-small-cell lung cancer (EURTAC): A multicentre, open-label, randomised phase 3 trial. Lancet Oncol (2012) 13(3):239–46. doi: 10.1016/S1470-2045(11)70393-X 22285168

[5] Yang JC Wu YL Schuler M Sebastian M Popat S Yamamoto N . Afatinib versus cisplatin-based chemotherapy for EGFR mutation-positive lung adenocarcinoma (LUX-lung 3 and LUX-lung 6): Analysis of overall survival data from two randomised, phase 3 trials. Lancet Oncol (2015) 16(2):141–51. doi: 10.1016/S1470-2045(14)71173-8 25589191

[6] Eck MJ Yun CH . Structural and mechanistic underpinnings of the differential drug sensitivity of EGFR mutations in non-small cell lung cancer. Biochim Biophys Acta (2010) 1804(3):559–66. doi: 10.1016/j.bbapap.2009.12.010 PMC2859716 20026433

[7] Mitchell RA Luwor RB Burgess AW . Epidermal growth factor receptor: Structure-function informing the design of anticancer therapeutics. Exp Cell Res (2018) 371(1):1–19. doi: 10.1016/j.yexcr.2018.08.009 30098332

[8] Paez JG Janne PA Lee JC Tracy S Greulich H Gabriel S . EGFR mutations in lung cancer: Correlation with clinical response to gefitinib therapy. Science (2004) 304(5676):1497–500. doi: 10.1126/science.1099314 15118125

[9] Carey KD Garton AJ Romero MS Kahler J Thomson S Ross S . Kinetic analysis of epidermal growth factor receptor somatic mutant proteins shows increased sensitivity to the epidermal growth factor receptor tyrosine kinase inhibitor, erlotinib. Cancer Res (2006) 66(16):8163–71. doi: 10.1158/0008-5472.CAN-06-0453 16912195

[10] Scheffler M Kobe C Zander T Nogova L Kahraman D Thomas R . Monitoring reversible and irreversible EGFR inhibition with erlotinib and afatinib in a patient with EGFR-mutated non-small cell lung cancer (NSCLC) using sequential [18F]fluorothymidine (FLT-)PET. Lung Cancer (2012) 77(3):617–20. doi: 10.1016/j.lungcan.2012.05.110 22726919

[11] Stopfer P Marzin K Narjes H Gansser D Shahidi M Uttereuther-Fischer M . Afatinib pharmacokinetics and metabolism after oral administration to healthy male volunteers. Cancer Chemother Pharmacol (2012) 69(4):1051–61. doi: 10.1007/s00280-011-1803-9 22200729

[12] Wind S Schnell D Ebner T Freiwald M Stopfer P . Clinical pharmacokinetics and pharmacodynamics of afatinib. Clin Pharmacokinet (2017) 56(3):235–50. doi: 10.1007/s40262-016-0440-1 PMC5315738 27470518

[13] Yang JC Shih JY Su WC Hsia TC Tsai CM Ou SH . Afatinib for patients with lung adenocarcinoma and epidermal growth factor receptor mutations (LUX-lung 2): a phase 2 trial. Lancet Oncol (2012) 13(5):539–48. doi: 10.1016/S1470-2045(12)70086-4 22452895

[14] Lau SCM Batra U Mok TSK Loong HH . Dacomitinib in the management of advanced non-Small-Cell lung cancer. Drugs (2019) 79(8):823–31. doi: 10.1007/s40265-019-01115-y 31069718

[15] Reckamp KL Giaccone G Camidge DR Gadgeel SM Khuri FR Engelman JA . A phase 2 trial of dacomitinib (PF-00299804), an oral, irreversible pan-HER (human epidermal growth factor receptor) inhibitor, in patients with advanced non-small cell lung cancer after failure of prior chemotherapy and erlotinib. Cancer (2014) 120(8):1145–54. doi: 10.1002/cncr.28561 PMC4164026 24501009

[16] Soria JC Felip E Cobo M Lu S Syrigos K Lee KH . Afatinib versus erlotinib as second-line treatment of patients with advanced squamous cell carcinoma of the lung (LUX-lung 8): An open-label randomised controlled phase 3 trial. Lancet Oncol (2015) 16(8):897–907. doi: 10.1016/S1470-2045(15)00006-6 26156651

[17] Mok TS Wu YL Ahn MJ Garassino MC Kim HR Ramalingam SS . Osimertinib or platinum-pemetrexed in EGFR T790M-positive lung cancer. N Engl J Med (2017) 376(7):629–40. doi: 10.1056/NEJMoa1612674 PMC6762027 27959700

[18] Mok TS Wu YL Papadimitrakopoulou VA . Osimertinib in EGFR T790M-positive lung cancer. N Engl J Med (2017) 376(20):1993–4. doi: 10.1056/NEJMc1703339 28514610

[19] Oxnard GR Hu Y Mileham KF Husain H Costa DB Tracy P . Assessment of resistance mechanisms and clinical implications in patients with EGFR T790M-positive lung cancer and acquired resistance to osimertinib. JAMA Oncol (2018) 4(11):1527–34. doi: 10.1001/jamaoncol.2018.2969 PMC6240476 30073261

[20] Santos ES Kaplan B Kirshner E Croft EF Sequist LV Chau M . Osimertinib for previously treated patients with advanced EGFR T790M mutation-positive NSCLC: Tolerability and diagnostic methods from an expanded access program. Oncol Ther (2018) 6(1):45–58. doi: 10.1007/s40487-018-0061-y 32700141 PMC7359991

[21] Papadimitrakopoulou VA Han JY Ahn MJ Ramalingam SS Delmonte A Hsia TC . Epidermal growth factor receptor mutation analysis in tissue and plasma from the AURA3 trial: Osimertinib versus platinum-pemetrexed for T790M mutation-positive advanced non-small cell lung cancer. Cancer (2020) 126(2):373–80. doi: 10.1002/cncr.32503 31769875

[22] Soria JC Ohe Y Vansteenkiste J Reungwetwattana T Chewaskulyong B Lee KH . Osimertinib in untreated EGFR-mutated advanced non-Small-Cell lung cancer. N Engl J Med (2018) 378(2):113–25. doi: 10.1056/NEJMoa1713137 29151359

[23] Ramalingam SS Vansteenkiste J Planchard D Cho BC Gray JE Ohe Y . Overall survival with osimertinib in untreated, EGFR-mutated advanced NSCLC. N Engl J Med (2020) 382(1):41–50. doi: 10.1056/NEJMoa1913662 31751012

[24] Kalemkerian GP Narula N Kennedy EB Biermann WA Donington J Leighl NB . Molecular testing guideline for the selection of patients with lung cancer for treatment with targeted tyrosine kinase inhibitors: American society of clinical oncology endorsement of the college of American Pathologists/International association for the study of lung Cancer/Association for molecular pathology clinical practice guideline update. J Clin Oncol (2018) 36(9):911–9. doi: 10.1200/JCO.2017.76.7293 29401004

[25] Rolfo C Mack P Scagliotti GV Aggarwal C Arcila ME Barlesi F . Liquid biopsy for advanced NSCLC: A consensus statement from the international association for the study of lung cancer. J Thorac Oncol (2021) 16(10):1647–62. doi: 10.1016/j.jtho.2021.06.017 34246791

[26] Rolfo C Mack PC Scagliotti GV Baas P Barlesi F Bivona TG . Liquid biopsy for advanced non-small cell lung cancer (NSCLC): A statement paper from the IASLC. J Thorac Oncol (2018) 13(9):1248–68 doi: 10.1016/j.jtho.2018.05.030. 29885479

[27] Bahce I Smit EF Lubberink M van der Veldt AA Yaqub M Windhorst AD . Development of [(11)C]erlotinib positron emission tomography for *in vivo* evaluation of EGF receptor mutational status. Clin Cancer Res (2013) 19(1):183–93 doi: 10.1158/1078-0432.CCR-12-0289. 23136193

[28] Bahce I Yaqub M Smit EF Lammertsma AA van Dongen GA Hendrikse NH . Personalizing NSCLC therapy by characterizing tumors using TKI-PET and immuno-PET. Lung Cancer (2017) 107:1–13 doi: 10.1016/j.lungcan.2016.05.025. 27319335

[29] Yaqub M Bahce I Voorhoeve C Schuit RC Windhorst AD Hoekstra OS . Quantitative and simplified analysis of 11C-erlotinib studies. J Nucl Med (2016) 57(6):861–6. doi: 10.2967/jnumed.115.165225 26848174

[30] van de Stadt Y . Quantification of [(18)F]afatinib using PET/CT in NSCLC patients: A feasibility study. EJNMMI Res (2020) 10(1):97. 32804306 10.1186/s13550-020-00684-4 PMC7431492

[31] van de Stadt EA Yaqub M Lammertsma AA Poot AJ Schuit RC Remmelzwaal S . Identifying advanced stage NSCLC patients who benefit from afatinib therapy using (18)F-afatinib PET/CT imaging. Lung Cancer (2021) 155:156–62. doi: 10.1016/j.lungcan.2021.03.016 33836373

[32] Group F-NBW . BEST (Biomarkers, EndpointS, and other tools) resource. Bethesda: Silver Springs (2016).

[33] Du B Wang S Cui Y Liu G Li X Li Y . Can (18)F-FDG PET/CT predict EGFR status in patients with non-small cell lung cancer? a systematic review and meta-analysis. BMJ Open (2021) 11(6):e044313. doi: 10.1136/bmjopen-2020-044313 PMC8190055 34103313

[34] Yin G Wang Z Song Y Li X Chen Y Zhu L . Prediction of EGFR mutation status based on (18)F-FDG PET/CT imaging using deep learning-based model in lung adenocarcinoma. Front Oncol (2021) 11:709137. doi: 10.3389/fonc.2021.709137 34367993 PMC8340023

[35] Bollineni VR Kramer GM Jansma EP Liu Y Oyen WJ . A systematic review on [(18)F]FLT-PET uptake as a measure of treatment response in cancer patients. Eur J Cancer (2016) 55:81–97. doi: 10.1016/j.ejca.2015.11.018 26820682

[36] Ullrich RT Zander T Neumaier B Koker M Shimamura T Waerzeggers Y . Early detection of erlotinib treatment response in NSCLC by 3'-deoxy-3'-[F]-fluoro-L-thymidine ([F]FLT) positron emission tomography (PET). PloS One (2008) 3(12):e3908. doi: 10.1371/journal.pone.0003908 19079597 PMC2592703

[37] Iqbal R Kramer GM Frings V Smit EF Hoekstra OS Boellaard R . Validation of [(18)F]FLT as a perfusion-independent imaging biomarker of tumour response in EGFR-mutated NSCLC patients undergoing treatment with an EGFR tyrosine kinase inhibitor. EJNMMI Res (2018) 8(1):22. doi: 10.1186/s13550-018-0376-6 29594931 PMC5874225

[38] de Jong IJ Pruim J Elsinga PH Vaalburg W Mensink HJ . 11C-choline positron emission tomography for the evaluation after treatment of localized prostate cancer. Eur Urol (2003) 44(1):32–8. doi: 10.1016/s0302-2838(03)00207-0 12814672

[39] Khan N Oriuchi N Zhang H Higuchi T Tian M Inoue T . A comparative study of 11C-choline PET and [18F]fluorodeoxyglucose PET in the evaluation of lung cancer. Nucl Med Commun (2003) 24(4):359–66. doi: 10.1097/00006231-200304000-00004 12673163

[40] Tian M Zhang H Oriuchi N Higuchi T Endo K . Comparison of 11C-choline PET and FDG PET for the differential diagnosis of malignant tumors. Eur J Nucl Med Mol Imaging (2004) 31(8):1064–72. doi: 10.1007/s00259-004-1496-y 15014903

[41] Hara T Inagaki K Kosaka N Morita T . Sensitive detection of mediastinal lymph node metastasis of lung cancer with 11C-choline PET. J Nucl Med (2000) 41(9):1507–13. 10994730

[42] Abdulla DSY Scheffler M Brandes V Ruge M Kunze S Merkelbach-Bruse S . Monitoring treatment response to erlotinib in EGFR-mutated non-small-cell lung cancer brain metastases using serial O-(2-[(18)F]fluoroethyl)-L-tyrosine PET. Clin Lung Cancer (2019) 20(2):e148–e51 doi: 10.1016/j.cllc.2018.10.011. 30528316

[43] Langen KJ Stoffels G Filss C Heinzel A Stegmayr C Lohmann P . Imaging of amino acid transport in brain tumours: Positron emission tomography with O-(2-[(18)F]fluoroethyl)-L-tyrosine (FET). Methods (2017) 130:124–34 doi: 10.1016/j.ymeth.2017.05.019. 28552264

[44] Varrone A Varnas K Jucaite A Cselenyi Z Johnstrom P Schou M . A PET study in healthy subjects of brain exposure of (11)C-labelled osimertinib - a drug intended for treatment of brain metastases in non-small cell lung cancer. J Cereb Blood Flow Metab (2020) 40(4):799–807. doi: 10.1177/0271678X19843776 31006308 PMC7168784

[45] Meng X Loo BW Jr. Ma L Murphy JD Sun X Yu J . Molecular imaging with 11C-PD153035 PET/CT predicts survival in non-small cell lung cancer treated with EGFR-TKI: A pilot study. J Nucl Med (2011) 52(10):1573–9. doi: 10.2967/jnumed.111.092874 21903741

[46] Sun X Xiao Z Chen G Han Z Liu Y Zhang C . A PET imaging approach for determining EGFR mutation status for improved lung cancer patient management. Sci Transl Med (2018) 10(431):443–55. doi: 10.1126/scitranslmed.aan8840 29515002

[47] Song Y Xiao Z Wang K Wang X Zhang C Fang F . Development and evaluation of (18)F-IRS for molecular imaging mutant EGF receptors in NSCLC. Sci Rep (2017) 7(1):3121. doi: 10.1038/s41598-017-01443-7 28600491 PMC5466683

[48] Yu JM Liu N Yang G Guo H Ma L Zhao S . 11C-PD153035 PET/CT for molecular imaging of EGFR in patients with non-small cell lung cancer (NSCLC). J Clin Oncol (2008) 26(15_suppl):3503. doi: 10.1200/jco.2008.26.15_suppl.3503 18640931

[49] Yu J Liu N Hu M Song X Xie L Meng X . Further evaluation of 11C-PD153035 as a molecular imaging probe for the assessment of the epidermal growth factor receptor status in non-small cell lung cancer patients. J Clin Oncol (2009) 27(15_suppl):3590. doi: 10.1200/jco.2009.27.15_suppl.3590

[50] Liu N Li M Li X Meng X Yang G Zhao S . PET-based biodistribution and radiation dosimetry of epidermal growth factor receptor-selective tracer 11C-PD153035 in humans. J Nucl Med (2009) 50(2):303–8. doi: 10.2967/jnumed.108.056556 19164239

[51] Memon AA Weber B Winterdahl M Jakobsen S Meldgaard P Madsen HH . PET imaging of patients with non-small cell lung cancer employing an EGF receptor targeting drug as tracer. Br J Cancer (2011) 105(12):1850–5. doi: 10.1038/bjc.2011.493 PMC3251890 22095231

[52] Cochet A Isambert N Foucher P Bertaut A Berthet C Coudert BP . Phase 0/1 of positron emission tomography (PET) imaging agent [18F]-ODS2004436 as a marker of EGFR mutation in patients with non-small cell lung cancer (NSCLC). J Clin Oncol (2018) 36(15_suppl):e24184. doi: 10.1200/JCO.2018.36.15_suppl.e24184

[53] Petrulli JR Zheng M Huang Y Nabulsi NB Goldberg SB Contessa JN . Evaluation of quantitative modeling methods in whole-body, dynamic [(11)C]-erlotinib PET. Am J Nucl Med Mol Imaging (2021) 11(2):143–53. PMC8165727 34079641

[54] Philippe Genne CB Raguin O Chalon S Tizon X Serriere S . Preclinical proof of concept for the first nanocyclix TKI-PET radiotracer targeting activated EGFR positive lung tumors. Cancer Res (2017) 77. doi: 10.1158/1538-7445.AM2017-1875A

[55] Bos M Mendelsohn J Kim YM Albanell J Fry DW Baselga J . PD153035, a tyrosine kinase inhibitor, prevents epidermal growth factor receptor activation and inhibits growth of cancer cells in a receptor number-dependent manner. Clin Cancer Res (1997) 3(11):2099–106. 9815602

[56] Slobbe P Windhorst AD Stigter-van Walsum M Schuit RC Smit EF Niessen HG . Development of [18F]afatinib as new TKI-PET tracer for EGFR positive tumors. Nucl Med Biol (2014) 41(9):749–57. doi: 10.1016/j.nucmedbio.2014.06.005 25066021

[57] Solca F Dahl G Zoephel A Bader G Sanderson M Klein C . Target binding properties and cellular activity of afatinib (BIBW 2992), an irreversible ErbB family blocker. J Pharmacol Exp Ther (2012) 343(2):342–50. doi: 10.1124/jpet.112.197756 22888144

[58] Wang DD Lee VH Zhu G Zou B Ma L Yan H . Selectivity profile of afatinib for EGFR-mutated non-small-cell lung cancer. Mol Biosyst (2016) 12(5):1552–63. doi: 10.1039/C6MB00038J 26961138

[59] O'Connor JP Aboagye EO Adams JE Aerts HJ Barrington SF Beer AJ . Imaging biomarker roadmap for cancer studies. Nat Rev Clin Oncol (2017) 14(3):169–86. doi: 10.1038/nrclinonc.2016.162 PMC5378302 27725679

[60] Badawi RD Shi H Hu P Chen S Xu T Price PM . First human imaging studies with the EXPLORER total-body PET scanner. J Nucl Med (2019) 60(3):299–303. doi: 10.2967/jnumed.119.226498 30733314 PMC6424228

[61] Vandenberghe S Moskal P Karp JS . State of the art in total body PET. EJNMMI Phys (2020) 7(1):35. doi: 10.1186/s40658-020-00290-2 32451783 PMC7248164

[62] Badawi RD Karp JS Nardo L Pantel AR . Total body PET: Exploring new horizons. preface. PET Clin (2021) 16(1):xvii–xviii doi: 10.1016/j.cpet.2020.09.005. 33218609

[63] Cherry SR Badawi RD Karp JS Moses WW Price P Jones T . Total-body imaging: Transforming the role of positron emission tomography. Sci Transl Med (2017) 9(381) doi: 10.1126/scitranslmed.aaf6169. PMC5629037 28298419

[64] Cherry SR Jones T Karp JS Qi J Moses WW Badawi RD . Total-body PET: Maximizing sensitivity to create new opportunities for clinical research and patient care. J Nucl Med (2018) 59(1):3–12 doi: 10.2967/jnumed.116.184028. 28935835 PMC5750522

[65] Colclough N Chen K Johnstrom P Strittmatter N Yan Y Wrigley GL . Preclinical comparison of the blood-brain barrier permeability of osimertinib with other EGFR TKIs. Clin Cancer Res (2021) 27(1):189–201. doi: 10.1158/1078-0432.CCR-19-1871 33028591

[66] Bauer M Karch R Wulkersdorfer B Philippe C Nics L Klebermass EM . A proof-of-Concept study to inhibit ABCG2- and ABCB1-mediated efflux transport at the human blood-brain barrier. J Nucl Med (2019) 60(4):486–91. doi: 10.2967/jnumed.118.216432 30237210

[67] Verheijen RB Yaqub M Sawicki E van Tellingen O Lammertsma AA Nuijen B . Molecular imaging of ABCB1 and ABCG2 inhibition at the human blood-brain barrier using elacridar and (11)C-erlotinib PET. J Nucl Med (2018) 59(6):973–9. doi: 10.2967/jnumed.117.195800 29175983

